# Human leucocyte antigen class I‐redirected anti‐tumour CD4^+^ T cells require a higher T cell receptor binding affinity for optimal activity than CD8^+^ T cells

**DOI:** 10.1111/cei.12828

**Published:** 2016-11-14

**Authors:** M. P. Tan, G. M. Dolton, A. B. Gerry, J. E. Brewer, A. D. Bennett, N. J. Pumphrey, B. K. Jakobsen, A. K. Sewell

**Affiliations:** ^1^ Cardiff University School of MedicineCardiffUK; ^2^ Adaptimmune LtdOxonUK

**Keywords:** CD4^+^ T cells, immunotherapy, MHC class I, TCR affinity

## Abstract

CD4^+^ T helper cells are a valuable component of the immune response towards cancer. Unfortunately, natural tumour‐specific CD4^+^ T cells occur in low frequency, express relatively low‐affinity T cell receptors (TCRs) and show poor reactivity towards cognate antigen. In addition, the lack of human leucocyte antigen (HLA) class II expression on most cancers dictates that these cells are often unable to respond to tumour cells directly. These deficiencies can be overcome by transducing primary CD4^+^ T cells with tumour‐specific HLA class I‐restricted TCRs prior to adoptive transfer. The lack of help from the co‐receptor CD8 glycoprotein in CD4^+^ cells might result in these cells requiring a different optimal TCR binding affinity. Here we compared primary CD4^+^ and CD8^+^ T cells expressing wild‐type and a range of affinity‐enhanced TCRs specific for the HLA A*0201‐restricted NY‐ESO‐1‐ and gp100 tumour antigens. Our major findings are: (i) redirected primary CD4^+^ T cells expressing TCRs of sufficiently high affinity exhibit a wide range of effector functions, including cytotoxicity, in response to cognate peptide; and (ii) optimal TCR binding affinity is higher in CD4^+^ T cells than CD8^+^ T cells. These results indicate that the CD4^+^ T cell component of current adoptive therapies using TCRs optimized for CD8^+^ T cells is below par and that there is room for substantial improvement.

## Introduction

Cytotoxic CD8^+^ T cells scan peptides displayed by human leucocyte antigen class I (HLA‐I) molecules at the cell surface. This mechanism allows T cells to kill cells that are infected with pathogens or that show dysregulation of their normal gene expression. Harnessing T cells for cancer immunotherapy is highly topical, and has seen much recent success 1. One promising approach involves the transfer of tumour‐specific T cell receptors (TCRs) into patient T cells to redirect them into killing cancer cells prior to adoptive transfer 2, 3. A potential limitation of this approach is that naturally occurring tumour‐specific T cells tend to have low sensitivity for cognate peptide antigen as a result of bearing TCRs with weaker binding affinity than those used to recognize pathogen‐derived peptides 4, 5. As a result, there is much interest in enhancing the affinity of cancer‐specific TCRs in order to optimize their sensitivity for therapeutic use. Several studies have shown that the use of enhanced‐affinity TCRs can improve the recognition of tumour cells and optimized receptors for the HLA A*0201 (HLA A2)‐restricted NY‐ESO‐1_157–165_ and HLA A*0101 (HLA A1)‐restricted MageA3_161–169_ systems have been trialled in patients 6, 7, 8, 9, 10, 11, 12. To date, most work has focused on CD8^+^ T cells due to their capacity to directly destroy tumour cells. Nevertheless, it is believed widely that tumour‐specific CD4^+^ T cells also play a vital role in anti‐cancer immunity. Having a predominately T helper type 1 (Th1) cytokine phenotype, tumour‐specific CD4^+^ T cells may provide help in the proliferation, differentiation and maintenance of CD8^+^ T cells in the tumour microenvironment and act in synergy with local CD8^+^ T cells to control tumour growth, even though they may not necessarily have direct cytotoxic abilities 13, 14, 15, 16. It is also possible that tumour‐specific CD4^+^ T cells may promote epitope spreading by neutralizing the immunosuppressive effects of the tumour microenvironment. The lack of HLA class II (HLA‐II) expression on most tumours poses a significant obstacle to the generation of natural, on‐site tumour‐specific CD4^+^ T cell help. Hence, an attractive solution to this problem would be to engineer CD4^+^ T cells so that they can recognize HLA‐I restricted cancer peptides and provide help directly at the tumour site. Such HLA‐I redirected CD4^+^ T cells have been shown to perform a variety of effector functions, including potent cytokine production and tumour cell lysis 17, 18, 19, 20, 21, 22, 23. However, the CD8 co‐receptor, absent in CD4^+^ T cells, is known to provide important roles in T cell activation by stabilizing interactions of HLA‐I with the TCR and optimizing TCR‐mediated signalling via delivery of the protein tyrosine kinase Lck to the intracellular side of the TCR–CD3 complex 24, 25, 26. This lack of adaptation of CD4^+^ T cells for recognition of HLA‐I might mean that the optimal affinity and/or dwell time of HLA‐I‐restricted TCRs might differ when they are expressed in CD4^+^ and CD8^+^ T cells. We have demonstrated previously that CD8 controls T cell cross‐reactivity towards HLA‐I‐restricted peptides 27. Interaction of CD8 with MHC stabilizes the TCR–peptide major histocompatibility complex (pMHC) interaction 26, thereby allowing weaker ligands to be recognized. CD8 also recruits the key kinase Lck to the cytoplasmic side of the TCR–CD3 complex and is critical to functional signalling by weaker ligands 24. These two functions combine to allow many more ligands to be recognized in the presence of CD8 [27]. The greater cross‐reactivity of HLA‐I‐restricted TCRs in the presence of CD8 suggests that there may be more opportunity to enhance the affinity of such TCRs in CD4^+^ T cells before off‐target effects are observed due to cross‐recognition of self‐peptides 27. In support of this notion, experiments in the murine 2C TCR model system comparing TCRs of two distinct affinities, CD4^+^ T cells expressing an MHC class I‐restricted TCR with a 1000‐fold higher affinity (m33 TCR, K_D_ = 30 nM) than the wild‐type (wt) 2C TCR (K_D_ = 30 μM), were found to control tumour growth and persisted for longer periods in the mice, compared to similarly transduced CD8^+^ T cells, which were deleted *in vivo* soon after transfer 28, 29. In the human HLA A2‐restricted NY‐ESO‐1_157–165_ tumour system, transduced CD8^+^ T cells expressing TCRs with a binding dissociation constant (K_D_) of 84 nM were found to be cross‐reactive, while transduced CD4^+^ T cells only displayed off‐target effects at considerably higher affinities 30.

In this study we evaluated formally the optimal binding affinity of HLA‐I‐restricted TCRs in CD4^+^ and CD8^+^ T cells by using a range of high‐affinity TCRs specific for two well‐studied and therapeutically important HLA A2‐restricted tumour antigens, NY‐ESO‐1_157–165_ and gp100_280–288_. Our results confirm that the TCR affinity required for optimal CD4^+^ T cell effector function is higher than that required for CD8^+^ T cells, and show that CD4^+^ T cells expressing higher‐affinity TCRs displayed potent effector function.

## Materials and methods

### Peptides

All peptides were purchased from PeptideSynthetics (Peptide Protein Research Ltd, Bishops Waltham, UK) in lysophilized form and reconstituted in dimethylsulphoxide (DMSO) (Sigma‐Aldrich, Poole, UK) to a stock solution of 4 mg/ml in DMSO and divided into aliquots such that the number of freeze–thaw cycles was kept to a minimum. Working concentrations of peptides were made in RPMI supplemented with 100 U/ml penicillin (Life Technologies, Paisley, UK), 100 μg/ml streptomycin (Invitrogen, UK) and 2 mM L‐glutamine (Life Technologies). The peptides used in activation assays were SLLMWITQC (SLL, NY‐ESO‐1_157–165_ epitope) and heteroclitic peptide YLEPGPVTV (YLE, gp100_280–288_ epitope).

### T cells and target cell lines

HLA A*0201^+^ (HLA A2), HLA^null^ C1R cells 24, 31 and HLA A2^+^ T2 cells 32, 33 were cultured in RPMI supplemented with penicillin, streptomycin, L‐glutamine and 10% heat‐inactivated fetal calf serum (FCS) (Gibco, Paisley, UK) (R10 medium). T cells were maintained in R10 with 25 ng/ml interleukin (IL)‐15 (PeproTech EC, London, UK), 200 IU/ml IL‐2 (PeproTech EC) and 2.5% Cellkines (Helvetica Healthcare, Geneva, Switzerland).

### Generation of CD8^+^ and CD4^+^ T cell cultures for lentiviral transduction

Blood bags from anonymous healthy donors were obtained from the Welsh Blood Service (Pontyclun, UK). Lymphocytes were purified using lymphoprep (Axia‐Shield, Dundee, UK) and typed for HLA A2 by antibody staining. CD8^+^ and CD4^+^ T cells were selected positively by CD8 and CD4 microbeads, respectively, purified through a magnetic affinity cell sorting (MACS) MS column (Miltenyi Biotec GmbH, Bergisch Gladbach, Germany) and resuspended at 10^6^/well in R10 with IL‐15, IL‐2 and Cellkines. Cells were activated overnight with αCD3/αCD28 Dynabeads (Invitrogen) at a bead to cell ratio of 3:1 before lentiviral transduction.

### Lentivirus generation and transduction of CD8^+^ and CD4^+^ T cells

HLA A2^+^ primary T cells were transduced with lentivirus expressing TCRs bearing various affinities for HLA A2‐restricted tumour antigens NY‐ESO‐1_157–165_ (SLLMWITQC) and gp100_280–288_ (YLEPGPVTV). Wild‐type and high‐affinity TCR mutants for NY‐ESO‐1_157–165_ and gp100_280–288_ were generated in this study or as described previously 9, 30, 34. The panel of TCR lentiviral constructs and their biophysical data are presented in Table 1. The lentiviral transduction system utilized in these studies was kindly provided by James L. Riley (University of Pennsylvania, USA) and was described previously 9. Briefly, lentiviral vector plasmids bearing each TCR construct were combined with packaging plasmids pRSV.REV, pMDLg/pRRE and pVSG‐V before transfection of 293T/17 cells (ATCC, Manassas, VA, USA) using the Express‐in transfection reagent (Open Biosystems, Huntsville, AL, USA). Supernatant containing lentiviral particles was collected after 24‐ and 48‐h incubations and concentrated by ultracentrifugation. Activated primary CD4^+^ and CD8^+^ T cells (10^6^ in 1 ml) were transduced with 1 ml concentrated lentivirus. Following 3 days' incubation, the efficiency of transduction was determined by flow cytometry after staining with relevant tetramer and αVβ TCR antibody, and Dynabeads were removed by magnet on day 5 following transduction.

### Measurement of soluble factors by enzyme‐linked immunosorbent assay (ELISA)

T cells were resuspended at 10^6^/ml in RPMI supplemented with penicillin, streptomycin, L‐glutamine and 2% FCS (R2 media) overnight. Target cells were pulsed with peptide for 1 h and washed to remove unbound peptide before T cells were added at an effector : target ratio of 1:2. After overnight incubation, tissue culture plates were centrifuged briefly to pellet cells, and the culture supernatant was harvested for measurement of lymphokines macrophage inflammatory protein (MIP)−1β, interferon (IFN)‐γ, tumour necrosis factor (TNF)‐α and IL‐2 by ELISA, performed according to the manufacturer's protocol (R&D Systems, Weisbaden, Germany).

### Monoclonal antibodies, amine reactive dyes and tetrameric complexes

Directly conjugated monoclonal antibodies were obtained as follows: (i) αMIP‐1β‐phycoerythrin (PE), αCD107a‐PE and αCD69‐PE (BD Biosciences, Oxford, UK); (ii) αTCR Vβ 13.1‐fluorescein isothiocyanate (FITC) and αTCR Vβ 17‐FITC (Beckman Coulter, High Wycombe, UK); (iii) live/dead fixable Aqua dead stain (Molecular Probes, Invitrogen, UK); (iv) αCD3‐Pacific blue, αCD3‐PEcyanin 7 (Cy7), αCD4‐allophycocyanin (APC) H7, αCD8‐APC H7, αCD19‐Brilliant Violet 521, αIFN‐γ‐PECy7, αTNF‐α‐PerCPCy5.5, αIL2‐APC, αperforin‐Brilliant Violet 421 (clone D48) and αgranzyme B‐AlexFlour 647 (Biolegend, London, UK); and (v) αCD3‐PCP (Miltenyi Biotech). Soluble peptide‐MHCI (pMHCI) monomers were generated and biotinylated as described previously 26, 35 and tetramers for SLLMWITQC/HLA A2 and YLEPGPVTV/HLA A2 were produced by adding streptavidin conjugated with APC (Molecular Probes, Invitrogen) 35. The HLA type of lymphocytes isolated from blood bags was determined using directly conjugated αHLA A2‐FITC (Serotec, Oxford, UK).

### Intracellular cytokine staining

T cells were resuspended at 10^6^/ml in R2 media overnight. Target cells were pulsed with peptide for 1 h and washed to remove unbound peptide before adding T cell effectors at an effector : target ratio of 1:2 together with 1 μl/ml brefeldin A (Sigma‐Aldrich) and 0.35 μl/ml monensin (BD Biosciences). After 5 h of antigen stimulation, cells were washed and stained sequentially with the Aqua live/dead stain to exclude dead cells and then with antibodies for surface molecules (CD3 and CD19). Cells were fixed and permeabilized using the BD Cytofix/Cytoperm kit and stained intracellularly for CD4 or CD8, TCR‐Vβ, MIP‐1β, IFN‐γ, TNF‐α and IL‐2. Stained cells were acquired using BD facsdiva version 8 (BD Biosciences) on the BD FACS Canto II (BD Biosciences). Cell population gates were set using fluorescent minus one (FMO) staining controls. For TCR‐transduced cells, events were gated on relevant Vβ^+^CD4^+^ or Vβ^+^CD8^+^ events for effector function analysis after Boolean gating using FlowJo version 7.6.4 (Treestar Inc., Ashland, OR, USA). Non‐transduced cells were gated on CD3^+^CD4^+^ or CD3^+^CD8^+^ events. A representative gating strategy is shown in Supporting Information, Fig. S1.

### Degranulation assay

T cells were resuspended at 10^6^/ml in R2 media overnight. T2 target cells were pulsed with peptide for 1 h and washed to remove unbound peptide before adding T cell effectors at an effector : target ratio of 1:2 together with 1 μl/ml brefeldin A (Sigma‐Aldrich), 0.35 μl/ml monensin (BD Biosciences) and 10 μl/ml αCD107a‐PE. After 5 h of antigen stimulation, cells were washed and stained sequentially with the Aqua live/dead stain to exclude dead cells and then with antibodies for surface molecules (CD3 and CD19). Cells were fixed and permeabilized using the BD Cytofix/Cytoperm kit and stained intracellularly for CD4 or CD8, TCR‐Vβ, granzyme B and perforin. Stained cells were acquired and analysed as described above.

### Target cell lysis by ^51^chromium release (^51^Cr) assay

For chromium release assays, 2 × 10^3^ HLA A2^+^ T2 target cells per well were labelled with 30 μCi of ^51^Cr (Perkin Elmer Life Sciences, Waltham, MA, USA) per 10^6^ cells for 1 h at 37°C. Targets were cultured alone to determine spontaneous release and with Triton X‐100 (Sigma‐Aldrich) at a final concentration of 5% to determine total release. Peptides were added to assay plates at varying concentrations and T cells were plated out in a final volume of 200 μl of R10 at effector : target ratios of 5:1 before incubation for 4 h at 37°C. For each sample, 15 μl of supernatant was harvested after incubation and mixed with 150 μl of OptiPhase supermix scintillation mixture (PerkinElmer Life Sciences). Data were acquired using a liquid scintillator and luminescence counter (MicroBeta TriLux; PerkinElmer Life Sciences) with MicroBeta Windows Workstation software (PerkinElmer Life Sciences). Specific lysis was calculated according to the following formula: (experimental release – spontaneous release/total release – spontaneous release) × 100.

## Results

### Redirecting primary CD4^+^ T cells with enhanced affinity HLA‐I‐restricted tumour‐specific TCRs

**Figure 1:**
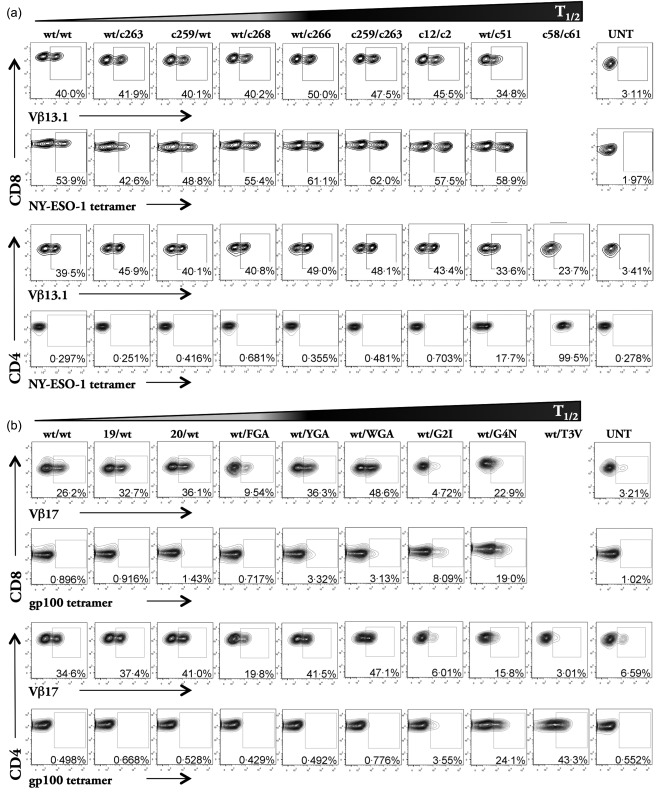
CD4^+^ T cells transduced with high affinity anti‐tumour T cell receptors (TCRs) stain with human leucocyte antigen (HLA)‐I tetramer in the absence of the CD8 co‐receptor molecule. HLA A2^+^ CD8^+^ and CD4^+^ T cells were transduced with NY‐ESO‐1 (a) and gp100 (b) TCRs and the transduction efficiency was assessed by TCR Vβ13.1/Vβ17 antibody staining, or by cognate tetramer. Cells were gated on live CD3^+^CD8^+^ or CD3^+^CD4^+^ populations. Values reflected here are from a single representative experiment of at least three independently performed experiments. UNT = non‐transduced cells.

Primary HLA A2^+^ CD4^+^ and CD8^+^ T cells were transduced with lentiviruses expressing the wild‐type and enhanced affinity HLA‐I‐restricted NY‐ESO‐1 and gp100 TCRs listed in Table 1. CD8^+^ T cells expressing the extremely high‐affinity NY‐ESO‐1 c58/c61 TCR (K_D_ = 26 pM) were not viable in culture and could not be studied, in contrast to the CD4^+^ T cells transduced with the same high‐affinity TCR. This is attributed probably to fratricide, as described previously 9. CD8^+^ and CD4^+^ T cells transduced with two gp100‐specific TCRs (wt/G2I, K_D_ = 402 nM; and wt/T3V, K_D_ = 26 nM) could not be stained with Vβ17 antibody, due probably to the location of the TCR mutations (Fig. 1). They were hence excluded from our study, as the lack of antibody staining made it difficult to assess the expression levels of wt/G2I and wt/T3V TCRs in functional assays.

**Table 1:** Biophysical data for the NY‐ESO157‐165 and gp100280‐288 T cell receptor systems

Specificity	TCR (α/β)	Half life (t_1/2_, s)	K_D_ (μM)	K_on_ (M^−1^s^−1^)	K_off_ (s^−1^)
NY‐ESO‐1_157‐165_	wt/wt	2.2	9.3	3.43 × 10^4^	3.1 × 10^−1^
(HLA‐A*0201)	wt/c263	9.6	1.13	6.36 × 10^4^	7.2 × 10^−2^
	c259/wt	19	0.730	4.87 × 10^4^	3.6 × 10^−2^
	wt/c268	33	0.16	1.28 × 10^5^	2.1 × 10^−2^
	wt/c266	40.8	0.28	5.82 × 10^4^	1.7 × 10^−2^
	c259/c263	74	0.12	7.7 × 10^4^	9.4 × 10^−3^
	c12/c2	173	0.45	9 × 10^3^	4 × 10^−3^
	wt/c51	495	0.025	5.4 × 10^4^	1.4 × 10^−3^
	c58/c61	68,100	2.6 × 10^−5^	5.7 × 10^5^	2.72 × 10^−5^
gp100_280‐288_	wt/wt	1.1	18.5	3.4 × 10^4^	6.35 × 10^−1^
(HLA‐A*0201)	19/wt	0.7	7.9	1.25 × 10^5^	9.9 × 10^−1^
	20/wt	0.88	4	1.97 × 10^5^	7.9 × 10^−1^
	wt/FGA	3.6	2.6	7.5 × 10^4^	1.94 × 10^−1^
	wt/YGA	5.7	1.9	6.5 × 10^4^	1.22 × 10^−1^
	wt/WGA	8.5	1.1	7.4 × 10^4^	8.2 × 10^−2^
	wt/G2I	10.2	0.402	2.75 × 10^5^	6.83 × 10^−2^
	wt/G4N	60.3	0.035	3.25 × 10^5^	1.15 × 10^−2^
	wt/T3V	89.2	0.026	3.04 × 10^5^	7.8 × 10^−3^

TCR, T cell receptor; K_D_, dissociation constant (affinity); K_on_, association rate; K_off_, dissociation rate; wt, wildtype. The HLA‐restriction for each system is indicated in brackets. TCRs shown in grey highlight were not included in the study because the cells were not viable or because the TCR mutations meant that they could not be tracked with Vβ antibody.

Primary CD4^+^ and CD8^+^ T cells transduced with all TCRs studied were stained with cognate pMHCI tetramer and antibody specific for the relevant TCR‐Vβ chain (Fig. 1). Apart from wt/G2I and wt/T3V TCRs from the gp100 panel, all cells stained with the relevant TCR‐Vβ antibody following transduction; staining levels in the NY‐ESO‐1 system varied between 24–50% and 9–37% in the gp100 system. CD8^+^ T cells transduced with all NY‐ESO‐1 TCRs including the wild‐type TCR could be stained with cognate SLLMWITQC/HLA A2 tetramers. In contrast, only CD4^+^ T cells with the wt/c51 and c58/c61 TCRs (K_D_s = 25 nM and 26 pM, respectively) could be stained with these reagents (Fig. 1a). Similar results were observed with the T cells transduced with gp100 TCRs, except that here CD8^+^ T cells with affinities lower than ∼2.6 μM (wt/FGA TCR) did not stain well with cognate YLEPGPVTV/HLA A2 tetramer (Fig. 1b). As with the NY‐ESO‐1 TCRs, only the CD4^+^ T cells expressing high‐affinity gp100 TCRs (wt/G2I, wt/G4N and wt/T3V) stained with gp100 tetramer. These results serve to highlight the important role that the CD8 co‐receptor plays in pMHCI multimer binding 26, 36.

CD4^+^ T cells transduced with all NY‐ESO‐1 TCRs responded to C1R targets transduced with HLA A*0201 (C1R‐A2) and pulsed with 100 nM cognate SLLMWITQC peptide but did not respond to untransduced A2^‐^C1R targets (Supporting information, Fig. S2). Production of MIP‐1β increased as far as the c259/263 TCR (K_D_ = 120 nM), with lower levels of production seen with higher‐affinity TCRs. Some background activation could be observed with substantially enhanced TCRs (Supporting information, Fig. S2). Overall, these data show that CD4^+^ T cells can be redirected to recognize an HLA‐I‐restricted peptide by TCR transduction with a relevant TCR and indicate that the optimal affinity/dwell time for recognition is higher than the wild‐type TCR isolated from a CD8^+^ T cell. We next set out to compare the optimum TCR affinity/half‐life in CD4^+^ and CD8^+^ T cells expressing NY‐ESO‐1 and gp100 TCRs.

### Comparison of TCR affinity optima in CD4^+^ and CD8^+^ T cells transduced with HLA‐I‐restricted TCRs

**Figure 2:**
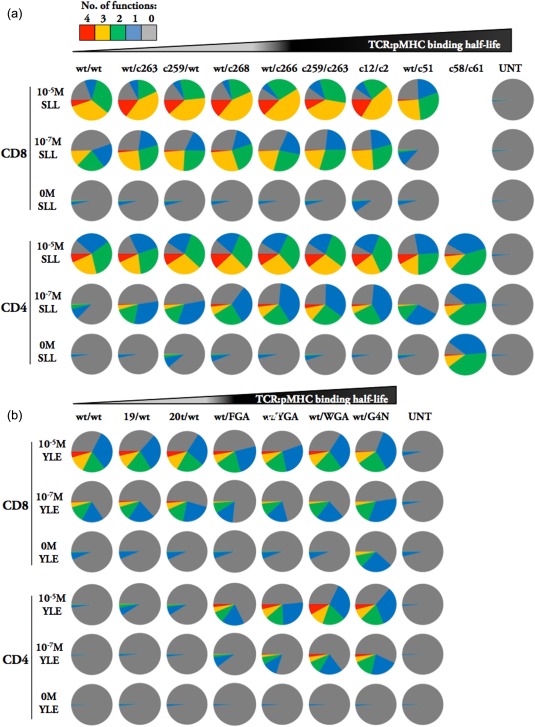
Human leucocyte antigen (HLA)‐I T cell receptor (TCR)‐transduced CD4^+^ T cells exhibit a polyfunctional effector profile upon peptide stimulation. Stimulation of HLA A2^+^ CD8^+^ and CD4^+^ T cells transduced with NY‐ESO‐1 (a) and gp100 (b) TCRs with or without the indicated concentrations of SLLMWITQC (SLL) and YLEPGPVTV (YLE) peptide, respectively, pulsed on HLA A2^+^ T2 target cells. Pie charts reflect proportion of cells exhibiting effector functions measured by intracellular staining with the area of each segment representing the proportion of cells displaying between 0 (grey) to 4 (red) functions. Cells are gated as described in Supporting information, Fig. S1. Effector functions measured were macrophage inflammatory protein (MIP)‐1β, interferon (IFN)‐γ, tumour necrosis factor (TNF)‐α and interleukin (IL)‐2 production. The biophysical data relating to the TCRs are described in Table 1. Values reflected here are from a single representative experiment of two independently performed experiments. UNT = non‐transduced cells.

T cells expressing similar amounts of HLA‐I‐restricted TCRs, as determined by CD3 and relevant Vβ staining, were incubated with cognate peptide‐pulsed T2 targets (HLA A*0201^+^) and their activation profiles were compared via intracellular cytokine staining (Fig. 2). Figure 2a shows effector function responses for cells transduced with NY‐ESO‐1 TCRs in the form of pie charts, as described previously 9. CD8^+^ T cells transduced with the second highest‐affinity TCR (wt/c51; K_D_= 25 nM) failed to respond well to 10^−7^ M cognate peptide, suggesting that this TCR was beyond optimal in this system, as noted previously 9. Simultaneous direct comparison with CD4^+^ T cells from the same individual showed some contrasting results, as CD4^+^ T cells expressing the c58/c61 TCR were viable. However, these cells responded to T2 targets in the absence of exogenous peptide, suggesting that they were able to see a self‐peptide in the context of HLA A2. Furthermore, CD4^+^ T cells transduced with the wild‐type NY‐ESO‐1 TCR failed to respond to target cells pulsed with 100 nM cognate peptide, highlighting the importance of the CD8 co‐receptor with natural‐affinity HLA‐I‐restricted TCRs as previously reported in the murine system 37. Results with CD4^+^ and CD8^+^ T cells transduced with the gp100 TCRs were similar, but more exaggerated (Fig. 2b). Here, CD8^+^ T cells expressing the highest‐affinity TCR (wt/3G4N; K_D _=_ _35 nM) reacted to targets in the absence of cognate YLEPGPVTV peptide, while CD4^+^ T cells transduced with the same TCR maintained peptide specificity. The wild‐type gp100 TCR (K_D_ = 18.5 μM) functioned well in CD8^+^ T cells, but CD4^+^ T cells expressing this TCR failed to respond even to 100 μM peptide. Indeed, even CD4^+^ T cells transduced with the affinity‐enhanced 20t/wt TCR (K_D_= 4 μM) failed to recognize peptide‐pulsed targets. Overall, these results suggest that CD4^+^ T cells require higher‐affinity TCRs with longer TCR‐peptide‐HLA dwell times for recognition of HLA‐I‐restricted peptides than similarly transduced CD8^+^ T cells. At the other end of the affinity spectrum, CD4^+^ T cells can tolerate higher‐affinity HLA‐I‐restricted TCRs than CD8^+^ T cells while still maintaining peptide specificity. In combination, these results suggest that the affinity of HLA‐I‐restricted TCRs required for optimal function is higher in CD4^+^ T cells than in CD8^+^ T cells.

### Effector function of CD4^+^ and CD8^+^ T cells transduced with HLA‐I‐restricted TCRs

**Figure 3:**
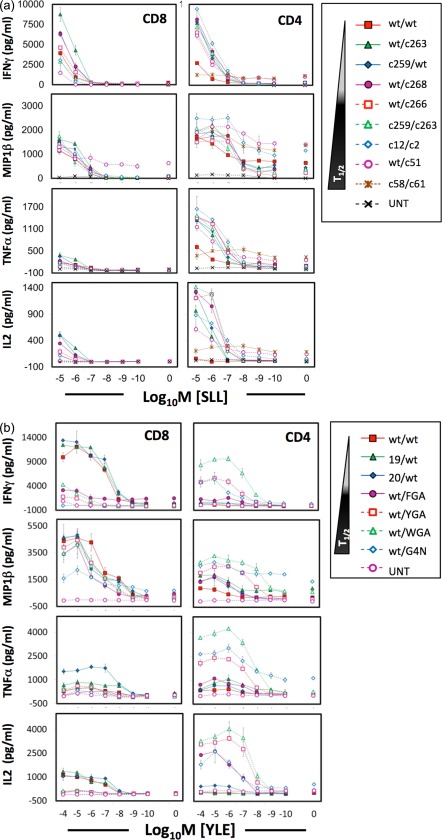
Human leucocyte antigen (HLA)‐I‐restricted NY‐ESO‐1 and gp100 T cell receptor (TCR)‐transduced CD4^+^ T cells exhibit a different cytokine profile compared to CD8^+^ counterparts in response to cognate antigen stimulation. CD8^+^ and CD4^+^ T cells transduced with the NY‐ESO‐1 (a) and gp100 (b) TCRs were stimulated with titrating concentrations of SLLMWITQC (SLL) and YLEPGPVTV (YLE) peptide, respectively, loaded on HLA A2^+^ T2 target cells. After overnight incubation, the culture supernatant was harvested for measurement of interferon (IFN)‐γ, macrophage inflammatory protein (MIP)‐1β, tumour necrosis factor (TNF)‐α and interleukin (IL)‐2 by enzyme‐linked immunosorbent assay (ELISA). The biophysical data of the higher‐affinity TCRs are described in Table 1. Values reflected here are from a single representative experiment of two independently performed experiments. UNT = non‐transduced cells.

We next examined how TCR‐transduced CD4^+^ and CD8^+^ T cells compared in terms of IFN‐γ, MIP‐1β, TNF‐α and IL‐2 production in response to a log‐fold titration of exogenous cognate peptide (Fig. 3). Naturally occurring tumour‐specific HLA‐II‐restricted CD4^+^ T cells and those engineered to express HLA‐I‐restricted tumour TCRs have been reported to exhibit a mainly Th1 phenotype, where cells typically produce IFN‐γ, TNF‐α and IL‐2 but not IL‐4, IL‐5 or IL‐10 [13–15,21]. In both the NY‐ESO‐1 and gp100 panels, CD4^+^ and CD8^+^ T cells made similar maximal amounts of IFN‐γ. Both cell types produced MIP‐1β in response to peptide, with the maximal response being slightly higher in CD4^+^ T cells with the optimal NY‐ESO‐1 TCR. In contrast, CD8^+^ T cells made more MIP‐1β than CD4^+^ T cells with the respective optimal gp100 TCR. CD4^+^ T cells were able to produce much higher levels of TNF‐α and IL‐2 than CD8^+^ T cells in response to peptide. In general, the wt/c263 TCR (K_D_∼1 μM) appeared optimal in CD8^+^ T cells and c12/c2 TCR (K_D_ = 0.45 μM) in CD4^+^ T cells with the NY‐ESO‐1 system. Similarly, the optimal‐affinity gp100 TCR was higher in CD4^+^ T cells (wt/G4N; K_D_= 35 nM) than in CD8^+^ T cells (20/wt; K_D_= 4 μM). Importantly, the natural‐affinity TCR was suboptimal in both systems and with all effector readouts. MIP‐1β was the most sensitive readout for CD8^+^ T cells with both TCRs in accordance with our previous studies, showing that this β‐chemokine provides the most sensitive readout for a wide range of CD8^+^ T cell clones 38, 39, 40. Indeed, background MIP‐1β production was observed with the wt/c51 TCR even in the absence of cognate peptide. Unexpectedly, MIP‐1β was also the most sensitive readout for bulk CD4^+^ T cells transduced with HLA‐I‐restricted TCRs. Similar results were observed when the HLA A2^+^ NY‐ESO‐1^+^ tumour cell line MEL624.38 was used as a target, confirming that CD4^+^ T cells could be reprogrammed to recognize tumour cells directly by producing Th1‐type cytokines (Supporting information, Fig. S3).

Overall, our findings support reports of tumour‐specific CD4^+^ T cells displaying a Th1 phenotype, although we did not formally rule out the production of Th2 type cytokines. Our results also show that redirected CD4^+^ T cells are able to exhibit effector functions which are absent in CD8^+^ T cells expressing the same antigen‐specific TCRs.

### HLA‐I‐redirected CD4^+^ T cells with enhanced TCRs exhibit efficient target lysis

**Figure 4:**
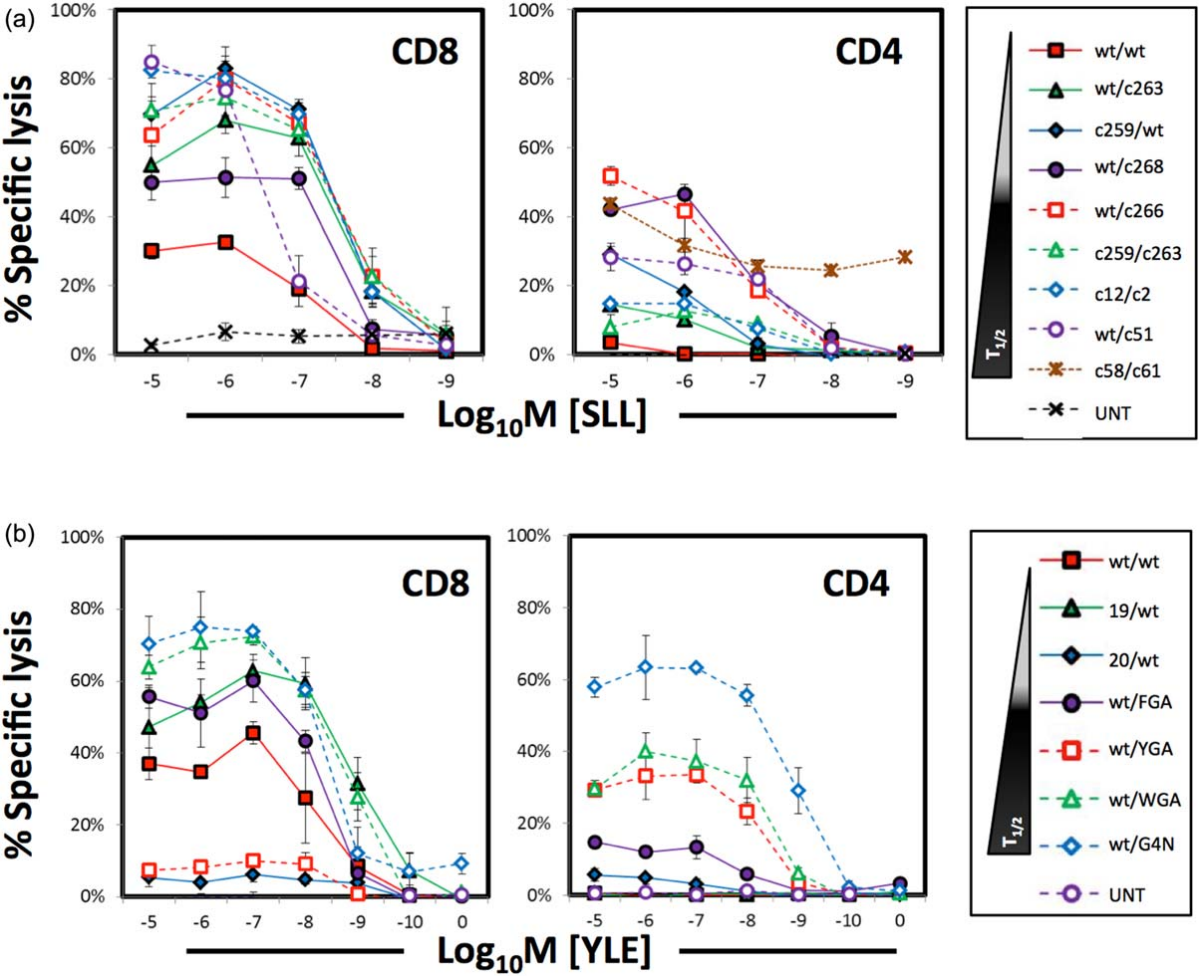
Human leucocyte antigen (HLA)‐I‐restricted CD4^+^ T cells are cytotoxic towards peptide‐pulsed targets. CD8^+^ and CD4^+^ T cells transduced with the NY‐ESO‐1 (a) and gp100 (b) T cell receptors (TCRs) were incubated with HLA A2^+^ T2 targets previously pulsed with ^51^chromium and loaded with titrating concentrations of SLLMWITQC (SLL) and YLEPGPVTV (YLE) peptide, respectively. Culture supernatant was collected after 4 h incubation and measured for ^51^chromium release. The biophysical data of the higher‐affinity TCRs are described in Table 1. Values reflected here are from a single representative experiment of at least two independently performed experiments. UNT = non‐transduced cells.

As described above, tumour‐specific CD4^+^ T cells play an important role in the provision of help to CD8^+^ T cells in the tumour environment. However, HLA‐I‐redirected CD4^+^ T cells might also be capable of direct, efficient tumour lysis. We next compared the ability of CD4^+^ T cells expressing both NY‐ESO‐1 and gp100 TCRs to lyse antigen‐bearing targets to that of CD8^+^ T cells (Fig. 4). As expected, transduced CD8^+^ T cells proved to be efficient killers of T2 targets pulsed with both cognate antigens. NY‐ESO‐1 TCR‐transduced CD4^+^ T cells could also lyse target cells, but they required a higher affinity TCR than was necessary for CD8^+^ T cell mediated lysis. The maximal specific lysis of ∼50% observed with NY‐ESO‐1 TCR‐transduced CD4^+^ T cells was lower than the ∼90% maximum seen with the wild‐type TCR in CD8^+^ cells. Our findings were similar in the gp100 system, although here, CD4^+^ T cells transduced with the highest‐affinity wt/G4N TCR tested (K_D_ = 35 nM) were almost as efficient at lysing targets as similarly transduced bulk CD8^+^ T cells.

### HLA‐I‐redirected CD4^+^ T cells with enhanced TCRs express granzyme B and perforin and degranulate in response to cognate antigen

**Figure 5:**
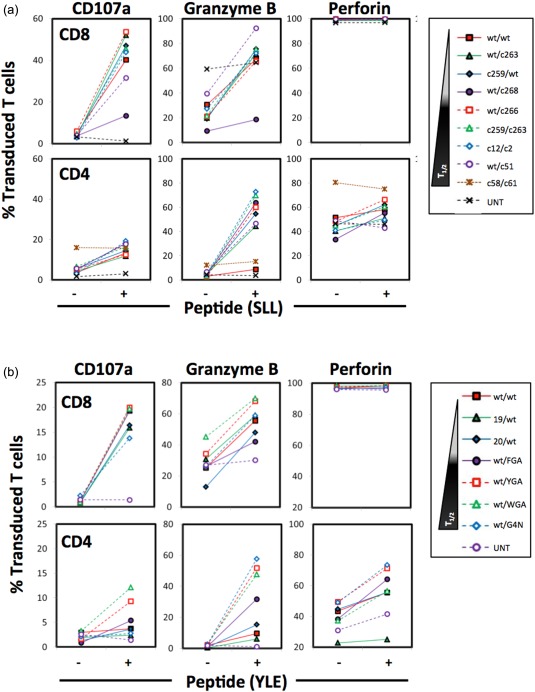
Human leucocyte antigen (HLA)‐I‐restricted CD4^+^ T cells kill via a lytic granule‐mediated mechanism. HLA A2^+^ CD8^+^ and CD4^+^ T cells transduced NY‐ESO‐1 (a) and gp100 (b) T cell receptors (TCRs) were incubated with HLA A2^+^ T2 target cells with or without 10^− 6 ^M SLLMWITQC (SLL) and YLEPGPVTV (YLE) peptide, respectively, for 5 h. They were then examined for surface CD107a and intracellular granzyme B and perforin expression by flow cytometry. Cells were gated on live CD3^+^CD8^+^ or CD3^+^CD4^+^ populations also expressing TCR Vβ13.1 or Vβ17 for NY‐ESO‐1 and gp100 TCRs, respectively. Non‐transduced cells (UNT) were gated on live CD3^+^CD8^+^ or CD3^+^CD4^+^ populations. Values reflected here are from a single representative experiment of three independently performed experiments.

Observations that engineered HLA‐I‐restricted CD4^+^ T cells could directly kill peptide‐pulsed or tumour targets have been reported previously, but the mechanism of target cell killing varies across studies 17, 18, 19, 21, 41. Hence, we next compared the mechanisms that HLA‐I‐redirected CD4^+^ and CD8^+^ T cells might be use to lyse target cells and examined the expression of CD107a, granzyme B and perforin in the transduced CD4^+^ and CD8^+^ T cells to determine whether the observed target cell killing could be mediated by lytic granule formation. We observed distinct and marked degranulation upon peptide activation for both the NY‐ESO‐1 and gp100 TCR‐transduced CD8^+^ T cells, as inferred by increased expression of CD107a (Fig. 5). Granzyme B production was also increased upon activation in both TCR‐transduced CD4^+^ and CD8^+^ T cells. Results between CD4^+^ and CD8^+^ T cells differed when perforin levels were examined. While all CD8^+^ transduced T cells expressed high levels of perforin, only ∼40% of CD4^+^ transduced T cells were found to express perforin, but this level increased after cells were incubated with HLA A2^+^ targets expressing cognate antigen indicating that perforin expression can be induced by antigen in some transduced CD4^+^ T cells. Our results indicate that CD4^+^ T cells redirected to HLA‐I‐restricted tumour antigens can kill tumour targets by a lytic granule‐mediated mechanism.

## Discussion

The use of affinity‐enhanced TCRs in adoptive cell transfer for cancer immunotherapy is proving promising in the clinic 42, 43, 44. Much attention has been focused upon tumour‐specific CD8^+^ T cells, due to the fact that they have the potential to directly kill tumour cells. However, it is recognized increasingly that tumour‐specific CD4^+^ T cells also play an instrumental role in tumour control. Several obstacles preclude easy exploitation of natural tumour‐specific HLA‐II‐restricted CD4^+^ T cells. First, such cells are typically present at very low frequency. Secondly, they tend to express relatively low‐affinity TCRs compared to anti‐pathogen TCRs, rendering them less sensitive to cognate antigen 5. Thirdly, very few natural tumour‐specific HLA‐II‐restricted peptide antigens have been defined. Finally, most tumours express HLA‐II poorly or not at all, thus limiting the potential for direct help at the tumour site. In addition, unlike tumour‐specific CD8^+^ T cells, anti‐tumour CD4^+^ T cells may lack direct cytotoxic abilities 16. In order to overcome these limitations, HLA‐I‐restricted tumour TCRs, usually of native affinity, have been expressed on CD4^+^ T cells such that they can be redirected to recognize HLA‐I‐restricted tumour antigens and contribute to the anti‐tumour immunity 17, 18, 19, 20, 21.

Here, we evaluated the impact of expressing a range of HLA‐I‐restricted tumour‐specific TCRs with different affinities for cognate antigen on CD4^+^ T cells and compared the functional efficacy of these redirected CD4^+^ T cells with CD8^+^ T cells expressing the same TCRs. Using two well‐studied HLA A2‐restricted tumour systems, CD4^+^ T cells transduced with enhanced affinity TCRs were shown to be potent effector cells in response to cognate antigen and exhibited direct tumour lysis.

There is much support in the literature for the synergistic effects of tumour‐specific CD4^+^ and CD8^+^ T cells within the cancer microenvironment 12, 14, 16, 17. In our study, polyclonal CD4^+^ primary T cells expressing high‐affinity HLA‐I‐restricted NY‐ESO‐1 and gp100‐specific TCRs produce high levels of TNF‐α and IL‐2. These cytokines were produced in low abundance by similarly transduced CD8^+^ T cells from the same individual(s). This finding is consistent with other studies showing that CD4^+^ T cells redirected to HLA‐I‐restricted tumour antigens produced more IL‐2 compared to transduced CD8^+^ T cells 19, 22. Indeed, others have reported that paracrine cytokine production can enhance the anti‐tumour functions of native or even high‐affinity CD8^+^ T cells by increasing CD8^+^ T cell accumulation at the tumour site, increasing CD8^+^ T cell tumour killing (e.g. by up‐regulation of granzyme and perforin) and CD8^+^ T cell proliferation 12, 14, 16, 17. Additional IFN‐γ release from CD4^+^ T cells may also up‐regulate HLA‐I and HLA‐II molecules on the tumour cell surface, which may result in the recruitment of more tumour‐specific CD8^+^ and CD4^+^ T cells to the disease site 45, 46.

Our data show that, in general, HLA‐I‐redirected CD4^+^ T cells required a TCR with an affinity higher than that needed by CD8^+^ T cells for optimal anti‐tumour effector function. CD4^+^ T cells transduced with optimal affinity TCRs were at least as sensitive to low antigen concentrations as CD8^+^ T cells. This finding contrasts with studies which reported that CD4^+^ T cells made to express HLA‐I‐restricted TCRs are markedly less sensitive than CD8^+^ T cells. For example, Xue *et al*. reported a 10‐fold reduction in peptide sensitivity when HLA‐I‐restricted Epstein–Barr virus (EBV) and cytomegalovirus (CMV)‐specific TCRs were expressed on CD4^+^ T cells; and when a HLA‐I‐restricted influenza nucleoprotein‐specific TCR was expressed on CD4^+^ T cells, Morris *et al*. found that CD4^+^ T cells were also less sensitive to peptide than CD8^+^ T cells 22, 23. Both these studies found that the sensitivity of transduced CD4^+^ T cells could be restored when the CD8 molecule was also transferred. Our results suggest that transduction of CD4^+^ T cells with enhanced‐affinity TCRs can largely bypass the need for the CD8 co‐receptor. These findings are supported by those of Chhabra *et al*., who expressed a high‐affinity HLA‐I‐restricted MART1 TCR (K_D_ in the nM range) on CD4^+^ T cells and reported that the transduced CD4^+^ T cells were as sensitive as CD8^+^ T cells in producing IFN‐γ in the presence of peptide‐pulsed targets 21. Also, co‐transfer of the CD8 molecule to CD4^+^ T cells expressing high‐affinity HLA‐I‐restricted TCRs has been shown to confer cross‐reactivity to self 30. In combination, the data to date suggest that HLA‐I‐restricted TCRs require different affinities and/or dwell times for optimal function in CD4^+^ T cells compared to CD8^+^ T cells.

In conclusion, we report that polyfunctional cytotoxic anti‐tumour CD4^+^ T cells can be generated through the transfer of enhanced affinity TCRs specific for HLA‐I‐restricted tumour antigens. The TCR affinity required for the optimal anti‐tumour functionality of CD4^+^ T cells is higher than that required for CD8^+^ T cells. CD4^+^ T cells redirected to recognize HLA‐I‐restricted antigens with optimal TCRs can respond to low peptide densities, making them attractive for immunotherapy, as tumour‐associated peptides are usually present at the cell surface at very low copy numbers of fewer than 50 copies per cell (47 and unpublished). As both CD4^+^ and CD8^+^ T cell responses are believed to be beneficial for tumour clearance, it could be advantageous to include HLA‐I‐redirected CD4^+^ T cells in adoptive cell transfer regimens in addition to CD8^+^ T cells. Our results suggest that the most beneficial therapy might involve engineered CD4^+^ and CD8^+^ T cells expressing different TCRs with the appropriate TCR : pMHC affinities to allow optimal, synergistic function of both T cell subsets.

## Author contributions

M. P. T. and G. D. performed the experiments. A. B. and N. P. generated reagents. A. K. S., B. J., M. P. T. and G. D. designed the study. A. K. S., B. J., M. P. T., J. B., A. G. and G. D. wrote the paper. A. K. S. provided financial support.

## Disclosure

There are no disclosures.

## Supporting information

Additional Supporting information may be found in the online version of this article at the publisher's web‐site:

**Fig. S1.** Representative gating strategy used in intracellular cytokine staining experiments to assess T cell polyfunctionality. Briefly, T cells were incubated with antigen and target cells after which surface and intracellular staining was performed. Lymphocytes were identified by their scatter profiles, and live CD3^+^ cells were identified after excluding aggregates and Aqua^+/^CD19^+^ cells. CD8/4^+^ Vβ13.1^+^ T cells [here indicating cells expressing NY‐ESO‐1 T cell receptor cells (TCRs)] were identified and cells producing interferon (IFN)‐γ, tumour necrosis factor (TNF)‐α, interleukin (IL)‐2 and macrophage inflammatory protein (MIP)‐1β were identified after Boolean gating. Polyfunctionality for non‐transduced CD8^+^ and CD4^+^ T cells was performed by gating on CD3^+^CD8 or CD3^+^CD4^+^ cells as a total population.

**Fig. S2.** CD4^+^ T cells expressing high‐affinity T cell receptors (TCRs) recognizing NY‐ESO‐1_157–165_ tumour antigen respond to peptide (SLLMWITQC, SLL) stimulation in the context of HLA‐I. CD4^+^ T cells transduced with the panel of NY‐ESO‐1_157–165_ TCRs were activated with human leucocyte antigen (HLA)‐A2^+^C1R target cells (A2^+^C1R) or HLA^null^ C1R cells (A2^‐^C1R) which were either pulsed with 10^−7 ^M SLL peptide or not. After overnight incubation, culture supernatant was harvested and the concentration of MIP‐1β was determined by enzyme‐linked immunosorbent assay (ELISA). UNT = non‐transduced cells.

**Fig. S3.** CD4^+^ T cells expressing NY‐ESO‐1 T cell receptors (TCRs) respond to a melanoma tumour cell line. CD8^+^ and CD4^+^ T cells expressing NYESO‐1 TCRs were incubated with or without the NY‐ESO‐1^+^ melanoma cell line MEL624.38 (MEL624) at the effector (E) to target (T) ratio of 5:1. After overnight incubation, culture supernatant was collected and assayed for the presence of interferon (IFN)‐γ and interleukin (IL)‐2 by enzyme‐linked immunosorbent assay (ELISA). UNT = non‐transduced cells.
