# Supplementary figures and images for: Human leucocyte antigen class I‐redirected anti‐tumour CD4+ T cells require a higher T cell receptor binding affinity for optimal activity than CD8+ T cells
Source: Clin Exp Immunol. 2016 Nov 14;187(1):124–37. doi: 10.1111/cei.12828 (PMC5167017; doi:10.1111/cei.12828)

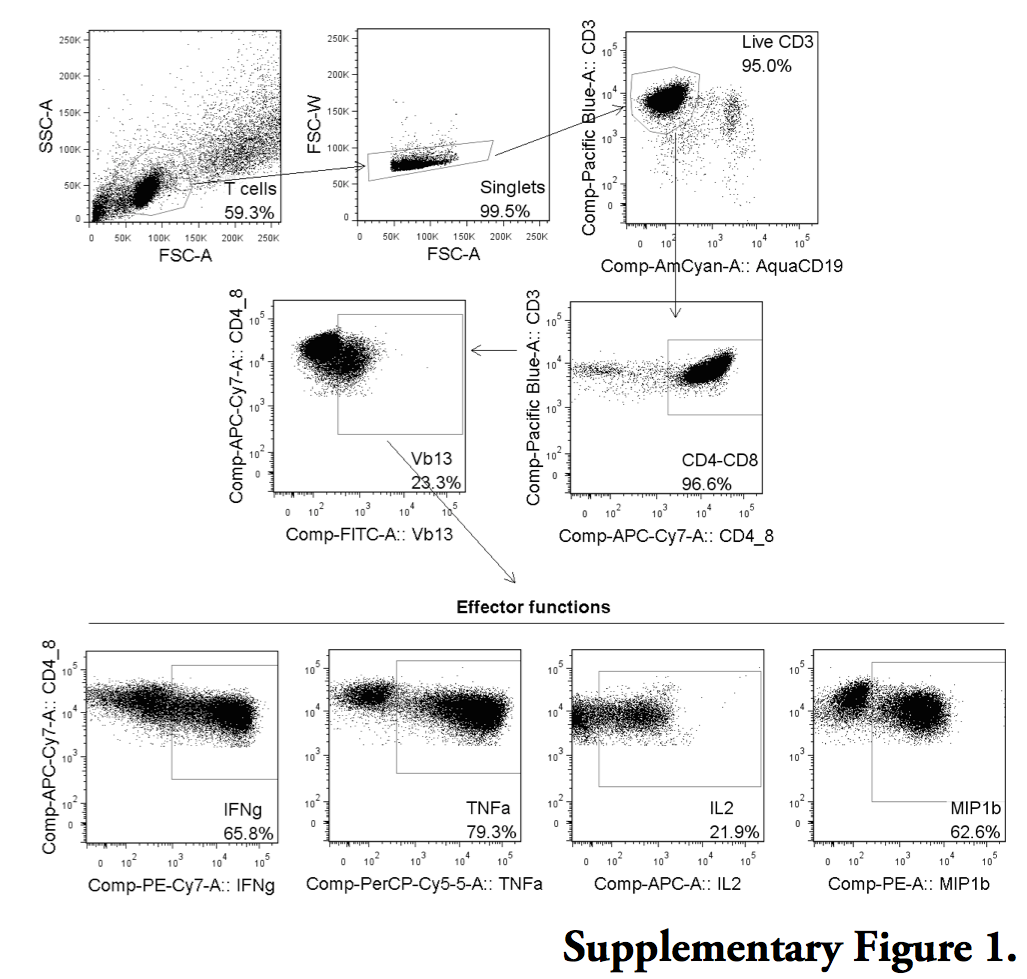

Supplement: Supplementary file 1 — Fig. S1. Representative gating strategy used in intracellular cytokine staining experiments to assess T cell polyfunctionality. Briefly, T cells were incubated with antigen and target cells after which surface and intracellular staining was performed. Lymphocytes were identified by their scatter profiles, and live CD3+ cells were identified after excluding aggregates and Aqua+/CD19+ cells. CD8/4+ Vβ13.1+ T cells [here indicating cells expressing NY‐ESO‐1 T cell receptor cells (TCRs)] were identified and cells producing interferon (IFN)‐γ, tumour necrosis factor (TNF)‐α, interleukin (IL)‐2 and macrophage inflammatory protein (MIP)‐1β were identified after Boolean gating. Polyfunctionality for non‐transduced CD8+ and CD4+ T cells was performed by gating on CD3+CD8 or CD3+CD4+ cells as a total population. [file CEI-187-124-s001.tiff]

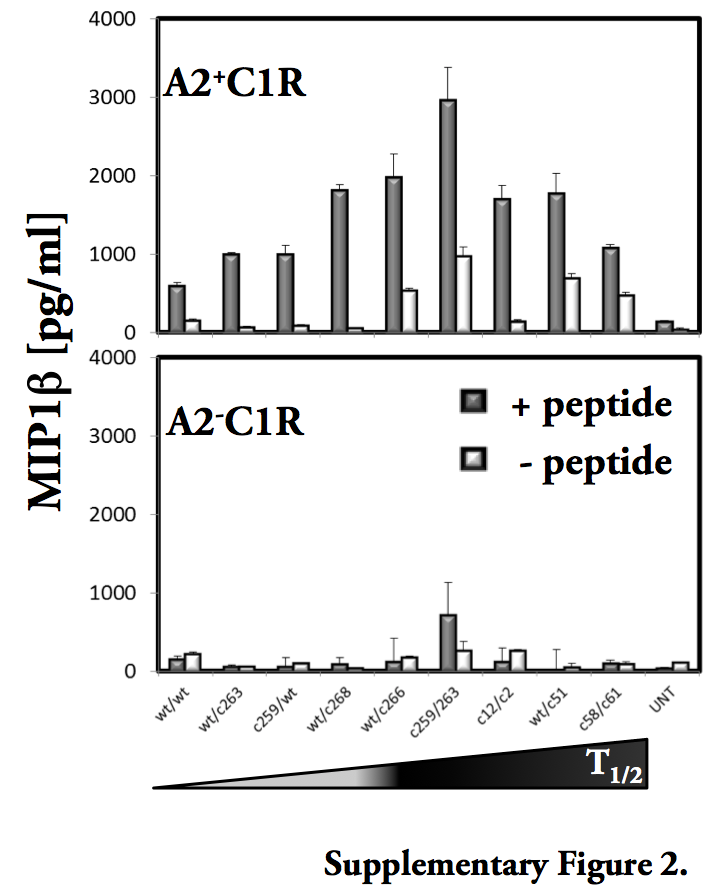

Supplement: Supplementary file 2 — Fig. S2. CD4+ T cells expressing high‐affinity T cell receptors (TCRs) recognizing NY‐ESO‐1157–165 tumour antigen respond to peptide (SLLMWITQC, SLL) stimulation in the context of HLA‐I. CD4+ T cells transduced with the panel of NY‐ESO‐1157–165 TCRs were activated with human leucocyte antigen (HLA)‐A2+C1R target cells (A2+C1R) or HLAnull C1R cells (A2‐C1R) which were either pulsed with 10−7 M SLL peptide or not. After overnight incubation, culture supernatant was harvested and the concentration of MIP‐1β was determined by enzyme‐linked immunosorbent assay (ELISA). UNT = non‐transduced cells. [file CEI-187-124-s002.tiff]

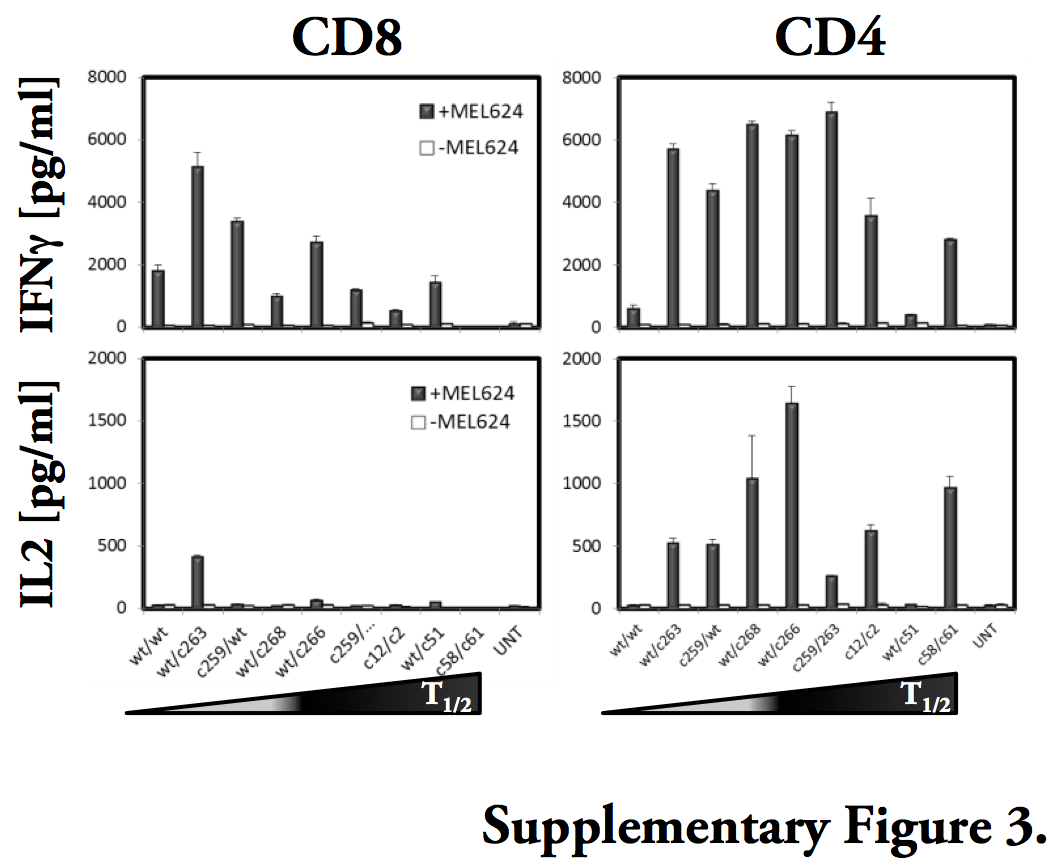

Supplement: Supplementary file 3 — Fig. S3. CD4+ T cells expressing NY‐ESO‐1 T cell receptors (TCRs) respond to a melanoma tumour cell line. CD8+ and CD4+ T cells expressing NYESO‐1 TCRs were incubated with or without the NY‐ESO‐1+ melanoma cell line MEL624.38 (MEL624) at the effector (E) to target (T) ratio of 5:1. After overnight incubation, culture supernatant was collected and assayed for the presence of interferon (IFN)‐γ and interleukin (IL)‐2 by enzyme‐linked immunosorbent assay (ELISA). UNT = non‐transduced cells. [file CEI-187-124-s003.tiff]
